# The Application Prospects and Challenges of Mesenchymal Stem Cell-Derived Exosomes in Spinal Cord Injury Repair

**DOI:** 10.2174/011574888X384819250823091635

**Published:** 2025-08-28

**Authors:** Yifeng Zhang, Cunxin Zhang, Kai Gao, Kang Li, Maoqing Fu, Chaoliang Lv

**Affiliations:** 1 Department of Graduate, Shandong First Medical University & Shandong Academy of Medical Sciences, Taian, Shandong 250117, China;; 2 Jining NO.1 People's Hospital Affiliated to Shandong First Medical University & Shandong Academy of Medical Sciences, Jining, China

**Keywords:** Mesenchymal stem cells, exosomes, spinal cord injury, regenerative repair, neural repair, cell apoptosis

## Abstract

Spinal cord injury (SCI) is a severe, disabling condition for which current treatments are largely insufficient in restoring neurological function. Despite advances in surgical and pharmacological interventions, no effective treatment currently exists to reverse neurological deficits caused by SCI. Mesenchymal stem cells (MSCs), especially human umbilical cord-derived MSCs (hucMSCs), have shown promise in tissue regeneration due to their multipotency and low immunogenicity. However, challenges such as low engraftment rates, tumorigenicity, and potential immune responses limit their clinical application. In recent years, mesenchymal stem cell-derived exosomes (MSC-Exos) have emerged as a promising therapeutic approach, demonstrating significant potential in SCI treatment. MSC-Exos exerts its therapeutic effects through mechanisms such as immune modulation, promotion of angiogenesis and axon regeneration, and reduction of blood-spinal cord barrier (BSCB) permeability. Furthermore, hucMSC-Exos demonstrate advantages in scalability, safety, and therapeutic efficacy, making them a promising cell-free approach for SCI repair. This review summarizes the biological properties of MSC-Exos, their roles in tissue injury repair, and their mechanistic contributions across different phases of SCI pathophysiology. Understanding these mechanisms will help pave the way for clinical translation of MSC-Exos as a novel and effective therapeutic strategy for SCI.

## 1 INTRODUCTION

 Spinal cord injury (SCI) is a severely disabling condition caused by trauma [1]. Epidemiological studies have reported that the annual incidence rate of SCI ranges from 15 to 40 cases per million people, with a notably higher prevalence among the elderly. In China, the incidence of SCI has shown a steady year-on-year increase. Between 1990 and 2019, the age-standardized incidence rate increased by 40.8% (95% UI: 32.9 - 49.1), indicating a significant upward trend in incidence when considering changes in the population's age structure [2]. Falls and traffic accidents remain the leading causes of SCI. Currently, clinical management of SCI mainly includes surgical intervention, pharmacological treatment, and postoperative rehabilitation. However, no effective treatment is available to restore the neurological dysfunction caused by SCI [3].

Therapeutic strategies for SCI are generally categorized into two main approaches: neuroprotection and regeneration [4]. With the advancement of biomedical technologies, some stem cell transplantation therapies, such as human bone marrow mesenchymal stem cell (hbMSCs) transplantation [5] and human umbilical cord mesenchymal stem cell (hucMSCs) transplantation [6], have demonstrated some potential in promoting spinal cord repair. However, the clinical application of traditional mesenchymal stem cell (MSC) transplantation remains limited by several critical challenges. These include poor cell survival and engraftment rates in the hostile post-injury microenvironment, the potential for immune rejection despite the relatively low immunogenicity of MSCs, and safety concerns such as tumorigenicity and the risk of microvascular embolism, particularly after systemic administration [7-9]. In light of these limitations, cell-free therapeutic strategies have garnered increasing interest. Among them, MSC-derived exosomes (MSC-Exos), as key mediators of MSC paracrine effects, offer several advantages, including superior tissue permeability, high biological stability, and minimal immunogenicity [10]. Notably, MSC-Exos are capable of crossing the blood-spinal cord barrier and exerting multiple biological functions, such as modulating immune and inflammatory responses, inhibiting apoptosis, and promoting neural regeneration [11, 12]. These properties position MSC-Exos as a promising alternative to traditional MSC transplantation, potentially overcoming its inherent limitations and offering a safer and more effective therapeutic strategy for SCI.

To systematically evaluate evidence on MSC-Exos for SCI repair, we searched PubMed and Web of Science (2010–2025) using combinations of "mesenchymal stem cell-derived exosomes," "spinal cord injury," and therapeutic keywords (*e.g*., "repair," "regeneration") with Boolean operators. We implemented strict inclusion criteria, focusing exclusively on original studies with explicit and rigorous characterization of exosomes to ensure the reliability of included evidence. Notably, approximately 40% of the included literature was published within the past five years, which allows us to incorporate recent advancements in the field while also considering valuable foundational research from earlier periods. This structured search and selection process formed the solid foundation for our analysis of the therapeutic mechanisms of MSC-Exos in SCI and the associated translational challenges. While MSC-Exos demonstrate significant promise in SCI repair, our review highlights that further research is still required to enhance mechanistic clarity and facilitate successful clinical translation.

## 2 MSC-EXOS AND TISSUE INJURY REPAIR

### 2.1 MSC-Exos

 MSCs, as pluripotent stem cells, possess not only the capacity for self-renewal but also the potential for multi-directional differentiation [13]. Their derivation from a wide range of tissues and organs further enhances their versatility [14]. Among various types of MSCs, hucMSCs exhibit superior proliferative ability and developmental flexibility plasticity, particularly owing to their origin from neonatal tissues [15]. Compared with other MSC sources, hucMSCs offer several advantages, including lower collection, less invasive, easier isolation procedures, higher cell yields, and improved gene transfection efficiency. These features make hucMSCs a particularly promising candidate for tissue injury repair [16]. Therefore, hucMSCs hold significant potential as an emerging therapeutic strategy for tissue regeneration, especially in the context of SCI repair [17].

Recent studies have increasingly demonstrated that MSCs exert their therapeutic effects through predominantly paracrine secretion, particularly *via* the secretion of extracellular vesicles (EVs) [18]. EVs play a critical role in mediating intercellular communication and regulating various physiological and pathological processes. Based on their size, biological properties, and synthesis processes, EVs are mainly classified into exosomes, microvesicles (MVs), and apoptotic bodies [19]. As early as 1983, Harding *et al*. [20] discovered small vesicles (<50 nm) secreted by reticulocytes during maturation that could transfer proteins to the extracellular space. Subsequently, Rose Johnstone introduced the term "exosome" to describe these vesicles [21]. Exosomes, as key mediators of cell–cell communication, carry nucleic acids, proteins, lipids, and metabolites, enabling them to induce complex biological effects. Their role in modulating the cellular microenvironment has garnered significant attention in recent years (Fig. **1**) [22, 23].

HucMSC-derived exosomes (hucMSC-Exos), produced through large-scale *in vitro* expansion of hucMSCs, combine several favorable attributes—such as ease of extraction, storage, and transport—with low immunogenicity and excellent biocompatibility [24, 25]. These properties make hucMSC-Exos a particularly attractive and practical option for therapeutic applications, including the treatment of central nervous system disorders like SCI.

### 2.2 MSC-Exos in Tissue Injury Repair

 MSCs not only migrate directly to damaged tissues for regeneration, but also modulate the local microenvironment through the secretion of bioactive factors. Among these, exosomes play a central role in the paracrine activity of MSCs, with promising potential for clinical applications. In myocardial injury, MSC-Exos contribute to cardiac repair following ischemic injury by reducing cardiomyocyte apoptosis, promoting cell proliferation, and enhancing angiogenesis. Notably, MSCs lacking exosomes secretion show limited therapeutic effect on cardiac function, suggesting that MSC-Exos are the primary therapeutic agents [26, 27]. In renal injury, MSC-Exos exert protective and regenerative effects by regulating cell proliferation, inhibiting apoptosis, and modulating the expression of growth-related genes (*CCNB1*, *CDK8*, *CDC6*) [28]. Zhang *et al*. [29] reported that hucMSC-Exos containing miR-20a alleviate liver ischemia/reperfusion (I/R) injury. Furthermore, miR-20a-containing MSC-Exos stimulate the expression of miR-20a target genes (such as *Beclin 1* and *FAS*) in hepatocytes, which are involved in apoptosis and autophagy and are related to the pathogenesis of liver I/R injury. In the context of SCI, MSC-Exos significantly reduce neuronal apoptosis and inflammation, promote vascular regeneration, limit lesion expansion, and facilitate functional recovery of the injured spinal cord [30, 31].

## 3 PATHOPHYSIOLOGY OF SCI

 The pathophysiology of SCI progresses through two interrelated phases: the primary injury phase and the secondary injury phase. These phases are tightly interconnected, with both contributing to the overall disease progression [32, 33]. The primary injury occurs immediately upon trauma, caused by direct mechanical forces leading to structural damage of the neural tissue, including neuronal, astrocytic, and oligodendrocytic injury, spinal cord hemorrhage, axonal rupture, and disruption of the BSCB [33, 34]. This structural damage sets the stage for a cascade of pathological events that extend into the secondary injury phase. Secondary injury typically unfolds over hours to weeks, marked by neuronal degeneration, cell apoptosis, inflammation, oxidative stress, excitotoxicity, and glial scar formation [32, 35].

 Intracellular calcium overload serves as a critical trigger for secondary injury in SCI [36-38]. The excessive influx of calcium ions disrupts cellular homeostasis, causing mitochondrial dysfunction and initiating the generation of reactive oxygen species (ROS) and reactive nitrogen species (RNS) [39]. These highly reactive molecules then attack cellular components such as lipids, proteins, and DNA, leading to widespread cellular damage, neuronal apoptosis, and necrosis [39]. Furthermore, the breach of the BSCB after the primary injury allows immune cells, including neutrophils, monocytes, macrophages, and T cells, to migrate to the injury site, activating innate immune pathways such as the NLRP3 inflammasome and triggering the release of pro-inflammatory cytokines like IL-1β, TNF-α, and IL-6, thereby exacerbating tissue damage [40-42]. Concurrently, activated astrocytes proliferate and secrete inhibitory extracellular matrix components, such as chondroitin sulfate proteoglycans (CSPGs), forming dense glial scars that impede axonal regeneration and functional recovery [43]. Oligodendrocyte loss leads to demyelination, severely affecting neuronal signal conduction [44]. In addition, apoptotic pathways, such as the Caspase-3 pathway and the imbalance between Bax and Bcl-2 proteins, as well as autophagy pathways, play significant roles in the secondary injury (Fig. **2A**) [45, 46].

SCI remains a critical unresolved medical issue, and efforts to translate preclinical findings into effective therapies are ongoing worldwide. Current treatment strategies, guided by the underlying pathophysiology of SCI, primarily aim to suppress inflammation, inhibit glial and fibrotic scar formation, promote remyelination, and enhance axon regeneration [47]. Despite promising preclinical data, none of these approaches has yet achieved satisfactory outcomes in clinical trials.

## 4 MSC-EXOS PROMOTE SCI REPAIR MECHANISMS

 MSC-Exos have been shown to traverse the compromised BSCB and deliver bioactive cargo—including miRNAs, proteins, and lipids—to the injury site. As illustrated in Fig. (**2B**), MSC-Exos exhibit stage-specific therapeutic effects by targeting acute oxidative stress and NF-κB signaling in the early phase, modulating macrophage polarization and preventing apoptosis during the subacute phase, and promoting axonal regeneration while limiting glial scar formation in the chronic phase [11]. By modulating immune and inflammatory pathways, promoting angiogenesis, supporting axonal regrowth, and restoring BSCB integrity, MSC-Exos act synergistically to enhance structural repair and functional recovery after SCI (Fig. **3** and Table **1**). Understanding these scientific issues will significantly advance the clinical application of MSC-Exos and provide safer and effective strategies for personalized biological treatments of human SCI.

### 4.1 MSC-Exos Inhibit Inflammatory Response

#### 4.1.1 Promote the Conversion of Macrophages and Microglia to Anti-Inflammatory Phenotype

 Inflammatory immune response is one of the most critical pathological processes and plays a key role in the prognosis of SCI. One of the core mechanisms by which MSC-Exos facilitate SCI repair is through modulating the polarization of macrophages and microglia. Following SCI, peripheral macrophages and resident microglia are rapidly activated and predominantly adopt a pro-inflammatory M1 phenotype. These cells release large quantities of inflammatory cytokines, including IL-1β, IL-6, TNF-α, and IFN-γ, thereby exacerbating tissue damage, inducing neuronal apoptosis, and impairing axonal regeneration [48, 49].

Microglia, one of the main resident cells in the central nervous system (CNS) following SCI, play a role in mediating neuroinflammation [50]. MSC-Exos have been shown to effectively shift this inflammatory microenvironment toward a reparative, anti-inflammatory state by promoting the transition of immune cells to an M2 phenotype. This process is largely mediated by Liu *et al*. [51] who found that hypoxia-enriched MSC-Exos, by transporting miR-216a-5p, suppressed the TLR4/NF-κB pathway and activated the PI3K/AKT pathway cascade (Fig. **3A**) [51]. As a result, M1 pro-inflammatory gene expression is downregulated, while M2 anti-inflammatory markers such as Arg-1, IL-10, and CD206 are upregulated [52]. Additionally, studies have demonstrated that MSC-derived exosomes containing miR-124 can directly inhibit neuroinflammation by promoting M2 polarization of microglia, suppressing mTOR signaling, and downregulating PDE4B gene expression (Fig. **3A**) [53].

#### 4.1.2 Inhibition of Lipopolysaccharide-Induced NF-κB Pathway Activation

 Under resting conditions, NF-κB signaling plays a key role in regulating essential cellular activities including proliferation, differentiation, migration, and survival. However, following SCI, pro-inflammatory factors such as cytokines, free radicals, and ROS can activate NF-κB, leading to inflammation and neurotoxic effects mediated by glial cells [54]. Treatment with bone marrow-derived MSC exosomes (BMSC-Exos) in SCI rats and lipopolysaccharide (LPS)-induced microglia has been shown to reduce pro-inflammatory cytokines levels and improve the Basso-Beattie-Bresnahan (BBB) score [55]. Following BMSC-Exos treatment, spinal cord tissue exhibited a significant increase in miRNA-181c expression, accompanied by inhibition of NF-κB (p-NF-κB) and downregulation of PTEN gene expressions (Fig. **3A**) [56]. These findings suggest that the miRNA-181c carried by BMSC-Exos can suppress microglia inflammation within spinal cord tissue, thereby exerting neuroprotective effects in SCI. In addition, other studies have demonstrated that MSC-Exos treatment significantly decreases p-NF-κB and p-IKBα levels in SCI models [57]. By directly inhibiting NF-κB activation, MSC-Exos effectively attenuate secondary inflammatory responses and reduce the inflammatory damage induced by LPS.

#### 4.1.3 Inhibition of Complement System Activation

 The complement system is a critical component of the immune system, playing a key role in enhancing immune responses. Under physiological conditions, complement activation is tightly regulated. However, alterations in the internal and external environments can lead to overactivation of the complement cascade, resulting in pathological dysfunction. In the early stages of SCI, a complex and multi-stage inflammatory response is initiated, which includes robust activation of the complement system. Studies have demonstrated that the classical complement pathway (C1q and C4), the alternative complement pathway (factor B), and the terminal pathway (C5b-9) are significantly activated within one day after SCI (Fig. **3A**). Complement protein-mediated immune responses are primarily observed in cell types such as neurons and oligodendrocytes that are vulnerable to damage [58].

In neurological diseases, MSC-Exos primarily affect microglia, which play a crucial role in immune regulation in the CNS [59, 60]. By interacting with microglia, MSC-Exos may modulate their activity, attenuating their sensitivity to complement activation and thereby preventing excessive complement cascade activation [57]. Notably, MSC-Exos, as a type of EVs, express EV markers such as CD55 and CD59 [61]. These markers are essential for preventing complement and coagulation factor activation, ensuring the widespread and stable distribution of MSCs-Exo in body fluids [61]. CD55, also known as decay-accelerating factor, binds to the C3 convertases of the classical and alternative complement pathways, accelerating the dissociation of their catalytic subunits. This action inhibits the deposition of C3b and subsequent complement activation, reducing the generation of inflammatory mediators [62]. CD59, or membrane attack complex inhibitory protein, blocks the assembly of the membrane attack complex (MAC, C5b-9), preventing its insertion into cell membranes and thus protecting cells from complement-mediated lysis [62, 63]. The presence of CD55 and CD59 on MSCs-Exo not only shields vulnerable cells such as neurons and oligodendrocytes from complement-induced damage but also maintains the integrity and functionality of MSCs-Exo during circulation (Fig. **3A**).

Furthermore, studies have indicated that complement system activation (*e.g*., *via* C3 and C5) is closely related to the NF-κB signaling pathway [64, 65]. Overactivation of the complement system can exacerbate inflammatory responses and further impair cellular function *via* the NF-κB pathway activation. MSC-Exos have been shown to suppress the synthesis and release of complement mRNAs, thereby reducing complement activity and indirectly inhibiting NF-κB pathway activation (Fig. **3A**) [57].

### 4.2 MSC-Exos Promote Angiogenesis

#### 4.2.1 Inhibition of Neuronal Cell Apoptosis

Glutamate (GLU) imbalance has been identified as a key contributor to the pathological outcomes of traumatic SCI. Excitotoxicity, triggered by excessive glutamate released from activated macrophages and microglia, plays a major role in secondary neuronal death following SCI [66], ultimately leading to neurological dysfunction. Elevated levels of GLU in the synaptic cleft induce neuronal neuronal apoptosis, which is a hallmark of SCI pathology. Liu *et al*. [67] reported that administration of BMSC-Exos in a rat model of traumatic SCI resulted in nearly a 70% reduction in neuronal apoptosis. In a glutamate-induced excitotoxicity model, BMSC-Exos exhibited significant neuroprotective effects. Specifically, BMSC-Exos treatment downregulated the expression of pro-apoptotic proteins Bax, caspase-3, and caspase-9, while upregulating the anti-apoptotic protein Bcl-2 [67]. In a separate study, Gu *et al*. [68] discovered that BMSC-Exos reduced neuronal apoptosis by promoting the expression of early autophagy-related proteins LC3IIB and Beclin-1, thereby contributing to improved functional recovery in rat following SCI (Fig. **3B**). Additionally, miRNA-21, a microRNA known for its role in regulating cell repair, proliferation, and apoptosis in tumor biology, also plays a critical role in central nervous system [69]. Studies have shown that MSCs-Exo transfected with miR-21 inhibit the expression of tumor suppressor factors (PTEN) and programmed cell death factor 4 (PDCD4), thereby enhancing neuronal cell viability and reducing apoptosis after SCI (Fig. **3B**) [70].

#### 4.2.2 Promotion of Endothelial Cell Proliferation and Migration

 Angiogenesis plays a vital role in the regeneration and repair processes following SCI. Injury-induced damage to the local vascular and the BSCB leads to inflammation and ischemia, resulting in extensive neural tissue damage within the spinal cord [71]. Insufficient vascularization limits nutrient delivery and waste removal, thereby hindering tissue repair and maintaining cells in a metabolically stressed state. Consequently, promoting angiogenesis has emerged as a promising therapeutic strategy for traumatic diseases and central nervous system injuries.

Vascular endothelial growth factor (VEGF) is a key regulator of angiogenesis. VEGF gradient promotes endothelial cell proliferation and migration, facilitating the formation of new tubular blood vessels [72]. Importantly, VEGF also acts as a crucial mediator of MSC-Exo-mediated proliferation and migration of human umbilical vein endothelial cells (HUVECs) [25]. Hypoxia, a major pathological feature following SCI, further exacerbates tissue damage. Notably, MSC-Exos preconditioned under hypoxic conditions exhibit enhanced angiogenic capacity, largely through increased expression of hypoxia-inducible factor 1-alpha (HIF-1α) and upregulation of VEGF in HUVECs, thereby promoting neovascularization in SCI models [73]. Systemic administration of MSC-Exos has been shown to enhance re-endothelialization in vein graft models and reduce intimal hyperplasia by upregulating the expression of pro-angiogenic genes in HUVECs [74]. *In vivo* studies further demonstrate that MSC-Exos promote angiogenesis and wound healing primarily through activation of the Wnt/β-catenin signaling pathway in endothelial cells (Fig. **3B**) [75].

### 4.3 MSC-Exos Promote Axonal Regeneration

#### 4.3.1 Inhibition of A1 Astrocyte Proliferation Reduces Glial Scar Formation in the Lesion Area

 Astrocytes are the most abundant glial cell type in the CNS and have a strong structural relationship with neurons through their wrapping of synaptic terminals, enabling signal transmission between glial cells and neurons [76]. During the secondary injury phase following SCI, astrocytes undergo a process known as reactive astrogliosis, characterized by their proliferation and hypertrophy. While reactive astrogliosis plays a dual role in CNS repair—offering both protective and inhibitory effects—it is frequently associated with the formation of glial scars, which can impede axonal regeneration [77-80]. Liddelow *et al*. [81] identified two types of reactive astrocytes: A1 astrocytes, which are induced by neuroinflammation and exhibit neurotoxic properties, and A2 astrocytes, which are ischemia-induced and exhibit neuroprotective effects. Notably, MSCs and MSC-Exos have been shown to selectively inhibit the proliferation and activation of A1 astrocytes. Intravenous injection of MSCs results in neuroprotective outcomes primarily mediated by exosomes, suggesting that MSC-Exos are the key active component responsible for modulating astrocyte behavior in the injured spinal cord [82].

 Repulsive axon guidance receptor Eph family is expressed in neurons, astrocytes, and meningeal fibroblasts following SCI, and is considered a key regulator that may influence SCI prognosis [83-85]. Among them, EphA4 is notably upregulated in astrocytes and neurons after SCI, plays a key role in inhibiting axonal regeneration and mediating reactive astrocyte proliferation [85]. Recent studies have shown that exosomal miR-532-5p can directly target and downregulate EPHA4, thereby interfering with its inhibitory effects on neural regeneration [86]. Moreover, administration of MSC-Exos in SCI mouse models leads to a marked suppression of neurotoxic A1 reactive astrocytes activation. By inhibiting toxic astrocyte proliferation, MSC-Exos treatment markedly reduces glial scar formation and enhances the capacity for neural regeneration and tissue repair (Fig. **3C**).

Additionally, MSC-Exos carrying specific miRNAs (*e.g*., miR-133b) play a significant role in promoting axonal growth. Studies have shown that MSC-Exos enriched with miR-133b can increase the expression of key axonal growth-associated proteins, including growth-associated protein 43 (GAP-43) and neurofilament proteins, thereby promoting axonal regeneration [87]. Mechanistically, miR-133b affects axonal regeneration-related signaling pathways by upregulating the expression of ERK1/2, STAT3, and CREB (Fig. **3C**) [87].

#### 4.3.2 Downregulation of the PTEN/mTOR Pathway

 Phosphatase and tensin homolog (PTEN) is an important inhibitory factor for axonal regeneration in the CNS [88]. By inhibiting the mTOR signaling pathway, PTEN suppresses protein synthesis and axonal regeneration. MSC-Exos have been shown to downregulate PTEN expression, thereby reactivating the mTOR pathway and promoting axonal regeneration [89]. Notably, MSC-Exos with PTEN gene silencing have been successfully delivered *via* the intranasal route to target the damaged area, promoting axonal regeneration [90]. Furthermore, exosomes derived from miR-26a-modified MSCs have been found to facilitate neurogenesis and reduce glial scar formation through the PTEN/AKT/mTOR signaling cascade (Fig. **3C**) [91].

### 4.4 MSC-Exos Reduce BSCB Permeability

#### 4.4.1 Reduction of Tight Junction Protein Loss in Endothelial Cells

BSCB is essential for maintaining the homeostasis of the spinal cord microenvironment, and its disruption is a hallmark pathological event following SCI [92]. Increased BSCB permeability after SCI permits the infiltration of inflammatory cells and harmful substances into the spinal cord parenchyma, thereby amplifying secondary injury [93]. A major contributor to this dysfunction is the downregulation of tight junction proteins, including occludin, claudin-5, and ZO-1, which are critical for maintaining endothelial cell integrity [94, 95].

Recent studies have highlighted the potential of MSCs and their secreted exosomes (MSC-Exos) in restoring BSCB integrity. MSC-Exos mitigate BSCB disruption through multiple mechanisms. First, miRNAs and proteins within MSC-Exos can upregulate the expression of tight junction proteins, enhancing the connectivity between endothelial cells and restoring BSCB barrier function (Fig. **3D**) [41, 96]. Second, MSC-Exos can inhibit inflammatory responses, reduce the release of inflammatory factors (IL-1β, IL-6, TNF-α, IFN-β), and minimize damage to the BSCB [11]. Additionally, MSC-Exos exert antioxidant properties, scavenging ROS and attenuating oxidative stress-induced DNA damage in endothelial cells [97].

#### 4.4.2 Inhibition of Pericyte Migration and Pyroptosis

 Pericytes are a type of cell located on the endothelial cells of microvessels and play a crucial role in the formation and maintenance of the BBB, BSCB, and neurovascular unit (NVU) in the CNS [98]. Disruption of the BSCB during SCI progression is a critical factor in causing neurological dysfunction and impeding recovery. Pericytes are essential for preserving BSCB integrity and homeostasis [98, 99], partly through the secretion of growth factors and nutrients that support surrounding cells and the extracellular matrix [100, 101]. Following SCI, pericyte loss, dysfunction, or aberrant migration from spinal cord microvessels leads to BSCB disruption, exacerbating inflammation and edema. Moreover, abnormal differentiation of pericytes may promote scar formation [102, 103], which impaired paracrine signaling can increase BSCB permeability, further worsening the injury response [103, 104]. Experimental studies have shown that under oxygen-glucose deprivation (OGD) *in vitro*, pericytes activate the NF-κB signaling pathway, with nuclear translocation of the p65 subunit and increased VEGF secretion [105]. Similar pathological upregulation of VEGF has been observed in ischemic stroke and CADASIL, where it contributes to BBB disruption *via* NF-κB pathway activation [105, 106]. These findings suggest that MSC-Exos may intervene in pericyte functions during SCI by regulating VEGF and NF-κB pathways, improving BSCB integrity, and promoting recovery [107].

 Pyroptosis, a form of programmed cell death dependent on caspase-1, caspase-4, caspase-5, and caspase-11, is characterized by cell membrane rupture and the release of pro-inflammatory cytokines, contributing to a vicious cycle of inflammation and tissue damage. Recent evidence indicates that MSC-Exos can suppress pyroptosis-related signaling pathways in pericytes, thus reducing inflammatory injury. Inhibition of pyroptosis has been shown to significantly improve functional recovery following SCI [108, 109]. Notably, transplantation of MSC-Exos significantly reduced spinal cord edema and caspase-1 expression, inhibited pericyte pyroptosis and IL-1β release, and ultimately preserved BSCB integrity (Fig. **3D**) [11]. Thus, pyroptosis may also contribute to pericyte loss and changes in BSCB permeability in SCI models.

## 5 CHALLENGES AND PROSPECTS

 Although MSC-Exos have demonstrated remarkable therapeutic potential in preclinical models of SCI, clinical studies in human patients remain extremely limited, representing a major translational barrier. A fundamental limitation lies in the incomplete understanding of their underlying mechanisms of action. While existing studies suggest that MSC-Exos exert neuroprotective and regenerative effects through mechanisms such as immune modulation, angiogenesis, inhibition of apoptosis, and preservation of the BSCB [56, 110, 111], the specific signaling pathways and cellular targets involved remain to be fully elucidated.

 The lack of clinical data remains a significant limitation in the current research on MSC-Exos for SCI. One major hurdle is the standardization and scalability of MSC-Exos production. The heterogeneity of exosomes—shaped by the origin of MSCs, culture conditions, and isolation techniques—poses challenges to batch-to-batch consistency and therapeutic efficacy [112, 113]. The development of robust, Good Manufacturing Practice (GMP)-compliant production protocols, alongside validated potency assays, is essential to ensure clinical-grade safety, quality, and reproducibility [114]. Furthermore, effective delivery of exosomes to spinal cord lesions remains technically challenging due to the anatomical and physiological complexity of the BSCB [115]. Innovative delivery strategies, including biomaterials-based carriers, surface modifications, and targeted delivery approaches, may help enhance exosome bioavailability and therapeutic localization. Although MSC-Exos are considered to have low immunogenicity, their long-term safety remains to be rigorously evaluated—particularly regarding the risks of immune activation, off-target effects, or interference with endogenous repair pathways [116]. In addition, clinical evidence is still scarce; most current data stem from animal models, and few human trials have investigated MSC-Exos specifically in the context of SCI. Nonetheless, ongoing trials in related neurological and inflammatory conditions, such as Alzheimer’s disease (NCT04388982) [117] and sepsis (NCT04850469) [118], are gradually building a foundation of safety and pharmacokinetic data that may inform future SCI applications.

Looking forward, continued research is needed to unravel the molecular mechanisms underpinning MSC-Exos activity, particularly in the areas of immune regulation, axonal regeneration, and vascular remodeling. Parallel efforts to optimize large-scale production, refine delivery platforms, and establish long-term safety profiles will be crucial for clinical translation. With ongoing interdisciplinary innovation and sustained clinical momentum, MSC-Exos hold substantial promise as a next-generation, cell-free therapeutic strategy for spinal cord repair—offering new hope for functional recovery in patients with SCI.

## CONCLUSION

 SCI remains a major global medical challenge, causing severe disability and placing a heavy burden on both society and the economy. This review summarizes recent progress in the study of of MSC-Exos for SCI repair. MSC-Exos have shown therapeutic potential by modulating immune system, inhibiting neuronal apoptosis and astrocyte proliferation, and promoting angiogenesis. These effects highlight their promise as a novel treatment strategy for SCI. Moreover, combining MSC-Exos with biomaterials can further enhance axonal regeneration by improving the injury microenvironment and supporting axonal growth. Although preclinical studies are encouraging, clinical evidence remains limited. Future research should aim to elucidate the underlying mechanism, conduct rigorous clinical trials, and optimize protocols for production, purification, and storage to ensure safety and feasibility. Overall, MSC-Exos represent a promising cell-free therapeutic approach with broad prospects for advancing SCI treatment.

## AUTHORS' CONTRIBUTIONS

Study conception and design: YZ and CZ; methodology: MF and CL; investigation: YZ, CZ, KG, KL and MF; visualization: YZ, CL and KG; draft manuscript: YZ and CZ. All authors reviewed the results and approved the final version of the manuscript.

## Figures and Tables

**Fig. (1):**
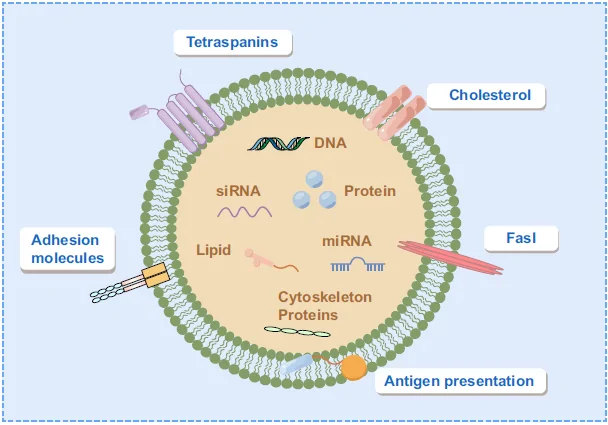
Schematic diagram of a small EV. The membrane contains tetraspanins, cholesterol, adhesion molecules, and FasL, which help with stability and cell communication. Inside, exosomes carry proteins, lipids, DNA, miRNA, and siRNA, allowing them to transfer signals between cells and regulate immune responses and tissue repair.

**Fig. (2):**
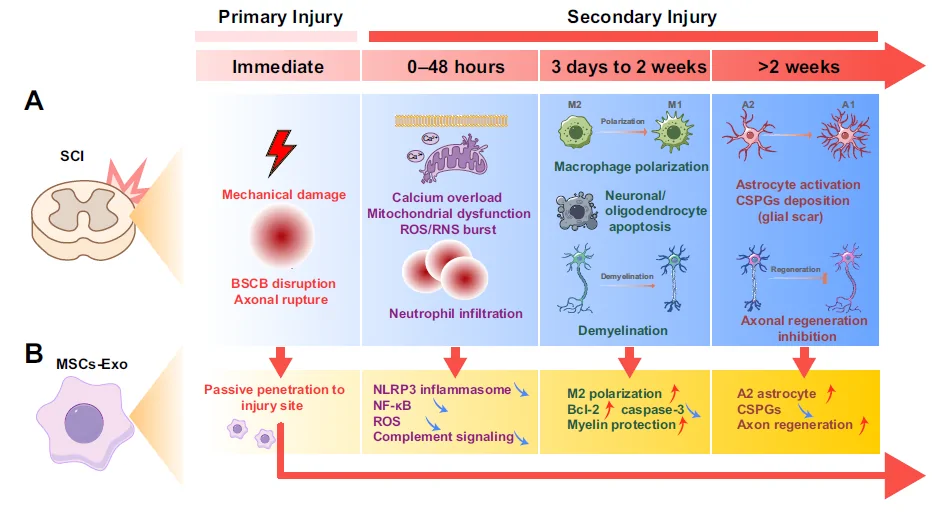
Pathophysiological timeline of SCI and intervention windows for MSC-Exos therapy. **(A)** SCI progression consists of primary injury (0 h, mechanical damage, and BSCB disruption) and secondary injury phases: acute (0–48 h, calcium overload, ROS/RNS release, inflammation), subacute (3 days–2 weeks, neuronal apoptosis, demyelination), and chronic (>2 weeks, astrocyte activation, axonal regeneration failure). **(B)** MSC-Exos exhibit stage-specific therapeutic effects by attenuating inflammation, suppressing apoptosis, and promoting myelin preservation and axonal regeneration.

**Fig. (3):**
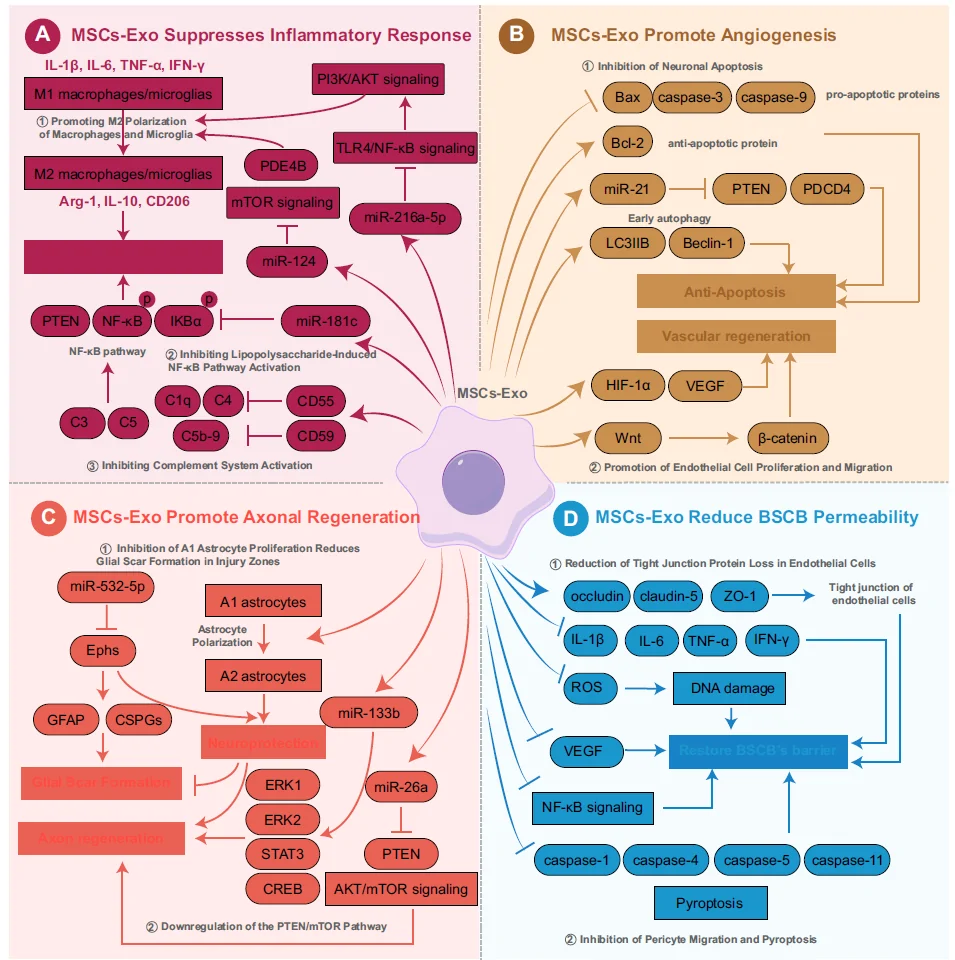
Physiological functions of MSC-Exos in SCI. **(A)** MSC-Exos suppress inflammatory response by regulating macrophage/microglia polarization (M1 to M2), modulating signaling pathways (PI3K/AKT, TLR4/NF-κB, mTOR) and miRNAs (*e.g*., miR-216a-5p), and inhibiting NF-κB activation, inflammation, and complement system activation. **(B)** MSC-Exos promote angiogenesis *via* regulating apoptosis-related proteins (Bax, caspase-3/9, Bcl-2), autophagy-associated molecules (LC3IIB, Beclin-1), and pathways (Wnt/β-catenin, HIF-1α/VEGF) for anti-apoptosis, endothelial cell function, and vascular regeneration. **(C)** MSC-Exos enhance axonal regeneration to shift astrocyte polarization (A1 to A2), suppress glial scar formation, and activate pathways (ERK1/2, STAT3, CREB, PTEN/mTOR) for neuroprotection and axon repair. **(D)** MSC-Exos reduce BSCB permeability by mitigating tight junction protein loss (occludin, claudin-5), modulating cytokines, oxidative stress, and regulating VEGF, NF-κB, and pyroptosis-related caspases to restore BSCB integrity.

**Table 1:** Mechanisms of MSC-exos in spinal cord injury (SCI) repair.

**Mechanism**	**Key Findings**	**Signaling Pathways/Components**	References
Immune Modulation	Promotes M1→M2 polarization of macrophages and microglia, reducing pro-inflammatory cytokines (IL-1β, IL-6, TNF-α, IFN-γ)	miR-216a-5p/TLR4/NF-κB, PI3K/AKT	[51]
	miR-124 in MSCs-Exo promotes M2 polarization by inhibiting mTOR signaling and downregulating PDE4B	miR-124 /mTOR, PDE4B	[53]
	miR-181c carried by MSCs-Exo suppresses p-NF-κB and PTEN expression, reduce microglial inflammation	miRNA-181c, p-NF-κB, p-IKBα, PTEN	[56, 57]
	Suppresses complement system activation *via* CD55/CD59, blocking membrane attack complex (MAC) formation.	CD55, CD59, complement proteins (C1q, C4, C5b-9)	[57, 61-63]
Angiogenesis	Reduces glutamate-induced excitotoxic neuronal apoptosis	Bax, caspase-3, and caspase-9, Bcl-2	[67]
	Upregulation of autophagy-related proteins (LC3II, Beclin-1) promotes neuroprotection	Autophagy (LC3IIB, Beclin-1)	[68]
	MSC-Exos carrying miR-21 inhibit PTEN and PDCD4, enhancing neuronal survival	miR-21/PTEN/ PDCD4	[69, 70]
	Hypoxia-preconditioned MSC-Exos increase HIF-1α and VEGF expression, enhancing angiogenesis	HIF-1α, VEGF	[73]
	Activates the Wnt/β-catenin pathway to promote neovascularization and wound healing	Wnt/β-catenin	[75]
Axonal Regeneration	MSC-Exos deliver miR-532-5p, which downregulates EphA4	miR-532-5p, EphA4 signaling	[86]
	MSC-Exos enriched with miR-133b increase expression of axonal regeneration markers: GAP-43 and neurofilament proteins	miR-133b, ERK1/2, STAT3, CREB	[87]
	MSC-Exos carrying miR-26a or PTEN-silenced exosomes promote neurogenesis and reduce glial scar *via* the PTEN/AKT/mTOR pathway	miR-26a, PTEN/AKT/mTOR	[90, 91]
BSCB Protection	Enhances the expression of tight junction protein loss (occludin, claudin-5), strengthening endothelial integrity	Occludin, Claudin-5, ZO-1	[41, 96]
	Reduces inflammatory cytokine release and suppresses inflammatory responses, protecting BSCB	IL-1β, IL-6, TNF-α, IFN-β	[11]
	Scavenges ROS and mitigates oxidative stress-induced DNA damage in endothelial cells	ROS, antioxidant pathway	[97]
	Regulates pericyte function by modulating VEGF and NF-κB signaling, thereby preserving vascular structure	VEGF, NF-κB signaling	[105, 107]
	MSC-Exos inhibit caspase-1 activation and IL-1β release in pericytes, preserving BSCB integrity and function	caspase-1, caspase-4, caspase-5, caspase-11, IL-1β	[11, 108, 109]

## LIST OF ABBREVIATIONS

BBB: Basso-Beattie-Bresnahan
BMSCsExo: Bone Marrow-Derived MSC Exosomes
BSCB: Blood-Spinal Cord Barrier
CADASIL: Cerebral Autosomal Dominant Arteriopathy With Subcortical Infarcts And Leukoencephalopathy
CNS: Central Nervous System
CSPGs: Chondroitin Sulfate Proteoglycans
EVs: Extracellular Vesicles
GAP-43: Growth-Associated Protein 43
GLU: Glutamate
GMP: Good Manufacturing Practice
hbMSCs: Human Bone Marrow Mesenchymal Stem Cells
HIF-1α: Hypoxia-Inducible Factor 1-Alpha
hucMSCs: Human Umbilical Cord-Derived MSCs
hucMSCsExo: Hucmscs-Derived Exosomes
HUVECs: Human Umbilical Vein Endothelial Cells
I/R injury: Ischemia/Reperfusion Injury
LPS: Lipopolysaccharide
MAC: Membrane Attack Complex
MSC-Exos: Mesenchymal Stem Cell-Derived Exosomes
MSCs: Mesenchymal Stem Cells
MVs: Microvesicles
NVU: Neurovascular Unit
OGD: Oxygen-Glucose Deprivation
PTEN: Phosphatase And Tensin Homolog
RNS: Reactive Nitrogen Species
ROS: Reactive Oxygen Species
SCI: Spinal Cord Injury
VEGF: Vascular Endothelial Growth Factor
